# Effect of Frost on the Different Metabolites of Two Mulberry (*Morus nigra* L. and *Morus alba* L.) Leaves

**DOI:** 10.3390/molecules28124718

**Published:** 2023-06-12

**Authors:** Lu Yang, Jiuyang Zhao, Shaoli Fan, Jinfa Liao, Yicun Chen, Yangdong Wang

**Affiliations:** 1 State Key Laboratory of Tree Genetics and Breeding, Chinese Academy of Forestry, Beijing 100091, China; yanglukitty127@163.com (L.Y.);; 2 Research Institute of Subtropical Forestry, Chinese Academy of Forestry, Hangzhou 311400, China; 3 Key Laboratory of Forest Resources and Utilization in Xinjiang of National Forestry and Grassland Administration, Xinjiang Academy of Forestry, Urumqi 830052, China; 4 Key Laboratory of Fruit Tree Species Breeding and Cultivation in Xinjiang, Urumqi 830052, China

**Keywords:** mulberry leaf, frost, metabolomics, DNJ alkaloid, flavonoid

## Abstract

Mulberry leaves are a well-known traditional Chinese medicine herb, and it has been observed since ancient times that leaves collected after frost have superior medicinal properties. Therefore, understanding the changes in critical metabolic components of mulberry leaves, specifically *Morus nigra* L., is essential. In this study, we conducted widely targeted metabolic profiling analyses on two types of mulberry leaves, including *Morus nigra* L. and *Morus alba* L., harvested at different times. In total, we detected over 100 compounds. After frost, 51 and 58 significantly different metabolites were identified in the leaves of *Morus nigra* L. and *Morus alba* L., respectively. Further analysis revealed a significant difference in the effect of defrosting on the accumulation of metabolites in the two mulberries. Specifically, in *Morus nigra* L., the content of 1-deoxynojirimycin (1-DNJ) in leaves decreased after frost, while flavonoids peaked after the second frost. In *Morus alba* L., the content of DNJ increased after frost, reaching its peak one day after the second frost, whereas flavonoids primarily peaked one week before frost. In addition, an analysis of the influence of picking time on metabolite accumulation in two types of mulberry leaves demonstrated that leaves collected in the morning contained higher levels of DNJ alkaloids and flavonoids. These findings provide scientific guidance for determining the optimal harvesting time for mulberry leaves.

## 1. Introduction

Mulberry is a tree species that is widely distributed in Asia, Europe, Africa, and other regions. It is characterized by its high growth rate, robust adaptability, and long-life cycle, making it a tree species with high ecological and economic benefits [1]. The fruits and leaves of mulberry contain abundant metabolites, which have high nutritional value. Mulberry not only has value as a food source but also serves as the primary food for silkworms and is an important raw material for silk production. Mulberry trees have hard and durable wood that can be used to manufacture furniture and building materials [2]. The most common types of mulberries in Mori are red, white, and *Morus alba* L. [3]. Red mulberry has been used by many Native American tribes to treat various diseases, and the juice of red mulberry is used to treat tinea [4]. White mulberry is known to thrive in various environmental conditions and climate changes. Many European countries, such as Turkey and Greece, have planted mulberry trees for food and the preparation of mulberry juice [5]. More than two thousand years ago, people in China raised silkworms with mulberry leaves, which gave rise to the world-famous Silk Road. Traditional mulberry silk production has had a profound influence on the development of human society [6]. In recent years, *Morus alba* L. has been found to not only have edible fruits but also to exhibit anti-diabetic and anti-cancer properties [7,8,9].

*Morus nigra* L., also known as medicinal mulberry, originated in Iran. After its introduction into China along the Silk Road, it became widely used for clearing heat and detoxifying, relieving coughs and dissolving phlegm, and treating colds, coughs, fevers, dysentery, and other ailments. In southern Xinjiang, Uighurs consider it a sacred fruit with a holy status [10]. Studies have reported that the main active components in the leaves of *Morus nigra* L. are 1-DNJ alkaloids, flavonoids, and phenols. It is known to have high nutritional value and exhibit antioxidant, hypoglycemic, glucose metabolism, lipid metabolism regulation, anti-inflammatory, antibacterial, and anticancer effects [11].

In many pharmacopoeias, medicinal mulberry leaves are harvested after the first frost, with frost mulberry leaves being preferred. This is because temperature is one of the main factors affecting the growth of mulberry leaves and metabolite synthesis [12,13]. Since the Ming and Qing dynasties, it has been believed that the best time to pick mulberry leaves is in the autumn or winter after frost. While the selection of frosted mulberry leaves was not initially proposed, it was discovered through practical experience and retained over time. In recent years, researchers have also found that the content of secondary metabolic components in mulberry leaves picked in different seasons is significantly affected by environmental temperature [14]. With the development of metabolomics and systems biology, researchers have begun to study the dynamic changes of metabolites in mulberry leaves before and after frost. For instance, Zou et al. found significant variations in phenol content and antioxidant activity of mulberry leaves from different varieties and picking times in southern China [15]. Xu et al. used metabolic pathway analysis and bioinformatics methods to speculate that lower temperatures would induce the expression of key enzyme genes in the flavonoid synthesis pathway and facilitate the accumulation of flavonoids [13]. Li et al. determined that there were differences in the alkaloid compound content of 711 mulberry varieties from 24 provinces in eastern China [16]. Despite numerous studies on the metabolites of mulberry leaves under different conditions, there is still a lack of systematic research on the dynamic changes of metabolites, particularly DNJ and flavonoid compounds, in mulberry leaves before and after frost.

Previous research has shown that the accumulation of active compounds, including flavonoids and their derivatives, in *Morus alba* L. leaves is significantly influenced by environmental factors such as temperature and harvest time [12,13]. However, the characteristics of changes in key metabolic components of mulberry leaves, especially those of *Morus nigra* L., are still not well understood. In this study, to address this research gap, we conducted a comparative metabolic profiling analysis of leaves from both *Morus alba* L. and *Morus nigra* L. before and after frost. This approach allowed us to identify the differences in the metabolic changes of these two types of mulberry leaves under the same environmental conditions. Our study offers significant guidance for the rational selection of optimal mulberry leaf harvesting times to effectively utilize key active compounds, including DNJ and flavonoid compounds found in the leaves. This knowledge can help increase the medicinal value and potential applications of mulberry leaves in various fields, including medicine and the food industry.

## 2. Results and Discussion

### 2.1. Detection of the Overall Metabolome of Mulberry Leaf and Evaluation of Data Quality

To investigate the impact of mulberry leaf harvest on the material accumulation pattern on different days after frost, we selected 138 samples from two different mulberry varieties, *Morus nigra* L. and *Morus alba* L., collected on 23 harvest days for metabonomic analysis. By conducting literature retrieval, standard comparison, public database matching, and metabolite profiling, we identified 150 different metabolites, including 16 types such as flavonoids and their derivatives, DNJ alkaloids, phenylpropane, lipids, anthocyanins, and amino acids (refer to Table S1 for details). This comprehensive approach allowed us to gain a deeper understanding of the metabolic changes in mulberry leaves over time, providing valuable insights into optimizing mulberry leaf harvesting practices and increasing the yield of key active compounds.

**Figure 1:**
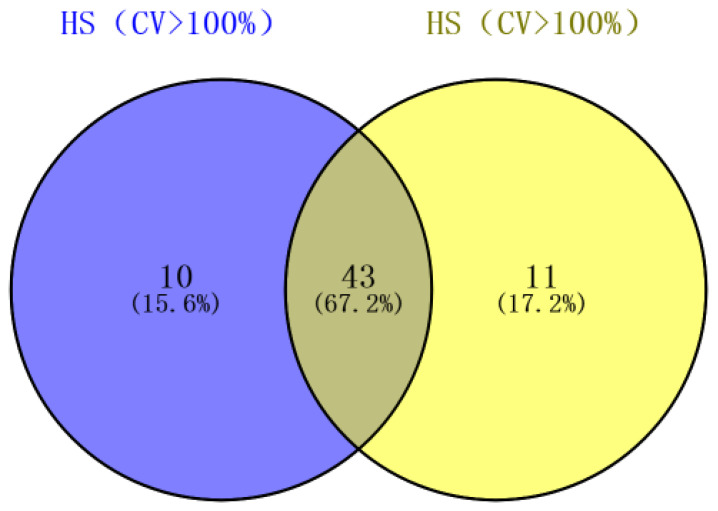
The metabolites with a CV value > 100% analyzed by a Venn diagram.

To comprehensively analyze the metabolome data (Table S2), statistical analysis was conducted on the content of detected metabolites in 138 samples. Results showed that 64.0% of metabolite coefficient variation (CV) exceeded 50%, with a proportion of 38.6% of metabolites having CV values greater than 100% (Table S1), indicating significant fluctuations in mulberry leaf metabolome data before and after the first frost event. These findings provide a robust foundation for subsequent analyses. For *Morus alba* L., the CV values of 35.5% and 36.0% of the metabolites at different growth stages exceeded 100%, respectively. Further analysis revealed that 67.2% of the metabolites exhibited CV values greater than 100% across both varieties, suggesting that the affected metabolites in the two varieties were largely similar across different growth and development stages. However, there were also specific metabolites that responded to changes between the two varieties (Figure 1).

### 2.2. Analysis of Differences in Metabolic Accumulation Patterns of Morus nigra L. and Morus alba L. before and after the Frost

**Figure 2:**
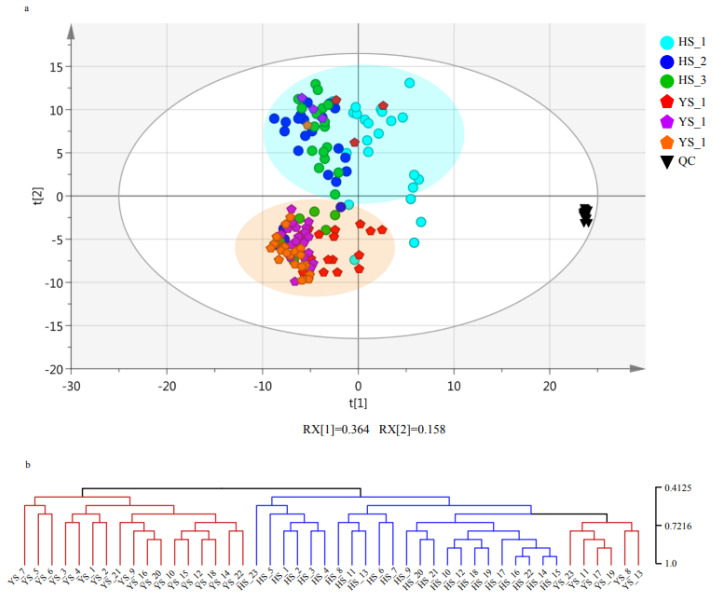
Analysis of the accumulation patterns of *Morus alba* L. and *Morus nigra* L. metabolites. (**a**) Principal component analysis (PCA) of metabolome in the 138 samples of *Morus nigra* L., *Morus alba* L. and QC. (**b**) Cluster dendrogram of metabolome.

In order to investigate the differences in metabolic accumulation patterns between *Morus nigra* L. and *Morus alba* L., we performed principal component analysis (PCA) on 138 tissue samples in this study. The results indicated that the metabolic accumulation patterns of the two species were significantly different and could be clearly separated into two distinct groups based on their metabolic profiles (Figure 2a). The first principal component, which accounted for 36.4% of the total variance, was found to mainly reflect the differences in metabolites between *Morus nigra* L. and *Morus alba* L. (Figure 2a). The clustering analysis tree diagram was consistent with PCA, with the 138 tissue samples divided into two main branches (Figure 2b).

Interestingly, we found that the metabolic accumulation patterns of *Morus nigra* L. exhibited significant changes on the day of frost, as demonstrated by the significant differences in metabolite profiles between tissues collected in the morning and evening of the frosting day (YS_11, YS_13, YS_17, and YS_19) compared to those collected at other times. Additionally, we observed that the time of day at which the leaves were collected on the frosting day (morning: YS_11 and YS_17; noon: YS_12 and YS_18; and evening: YS_13 and YS_19) had effects on the metabolic accumulation pattern of *Morus nigra* L. (Figure 2b).

**Figure 3:**
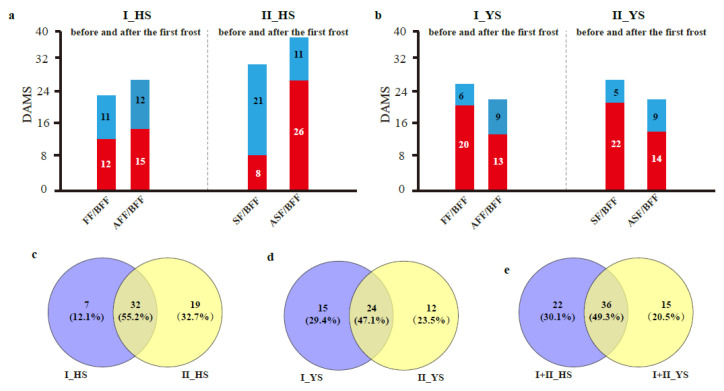
Analysis of differential accumulated metabolites (DAMS) in *Morus alba* L. (HS) and *Morus nigra* L. (YS) before and after two frost events. (**a**) DAMS was conducted for HS before and after the first and second frost events (I_HS and II_HS); (**b**) DAMS was conducted for YS before and after the first and second frost events (I_YS and II_YS); (**c**) Venn diagram analysis identified common differential metabolites in HS before and after the first and second frost events (I_HS and II_HS); (**d**) Venn diagram analysis identified common differential metabolites in YS before and after the first and second frost events (I_YS and II_YS). (**e**) Venn diagram analysis of shared DAMS between two varieties (HS and YS) before and after two frosts. “FF/BFF” represents DAMS analysis between the day of the first frost and the day before the first frost; “AFF/BFF” represents DAMS analysis between the day after the first frost and the day before the first frost; “SF/BFF” represents DAMS analysis between the day of the second frost and the day before the first frost; “ASF/BFF” represents DAMS analysis between the day after the second frost and the day before the first frost. Here, the abbreviation “FF” represents the day of the first frost, which includes samples YS_11, and “BFF” presents sample collected before the first Frost, including YS_10; The abbreviation “AFF” represents samples collected after the first frost event, which includes YS_14, and "SF" represents the day of second frost event, which includes YS_17. The abbreviation “ASF” represents samples collected after the second frost event, including YS_20.

We performed further analysis and quantification of differentially accumulated metabolites (DAMS) in *Morus alba* L. and *Morus nigra* L. before and after two frost events (Tables S3 and S4). Our findings revealed that *Morus alba* L. exhibited 58 DAMS before and after two frosts, while *Morus nigra* L. had 51 differential metabolites (Figure 3a–d). The number of common DAMS in HS after the first and second frost events was 33 (55.2%), whereas in YS it was 24 (47.1%) (Figure 3c,d). Nevertheless, after exposure to frost conditions, the shared differential metabolites between the two varieties constituted about 50% of the total changed metabolites (Figure 3e), indicating substantial differences in compounds affected by frost in the two varieties.

### 2.3. Dynamic Changes of Metabolite Contents of Morus nigra L. and Morus alba L. before and after Frost

**Figure 4:**
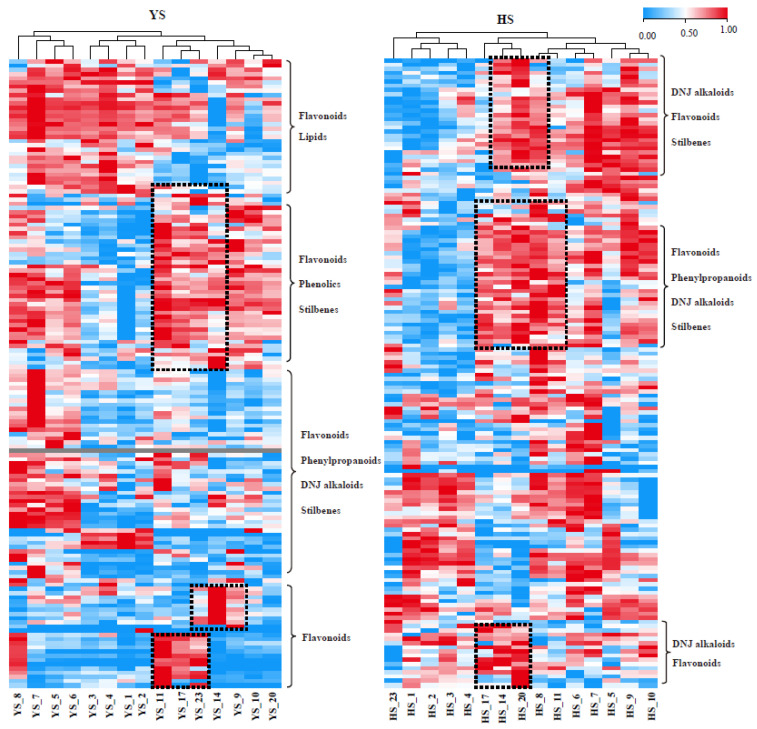
Heatmap of metabolites accumulation trend between *Morus nigra* L. and *Morus alba* L. after frost. Box is shown as significantly enriched metabolites after frost.

The analysis of the 150 metabolites present in the leaf tissues collected at different times revealed significant differences in the changes in metabolite content between *Morus nigra* L. and *Morus alba* L. after undergoing frost (Tables S3 and S4; Figure 3). After analyzing the dynamic changes of important metabolites in both varieties, it was found that in *Morus nigra* L., some flavonoids and stilbenes were significantly enriched after frost, while the content of certain flavonoids and most DNJ alkaloids considerably decreased. Conversely, after the first frost event, DNJ alkaloids, flavonoids, and stilbenes were significantly enriched in *Morus alba* L. Some flavonoids had the highest content before frost and gradually decreased over time (as shown in Figure 4).

**Figure 5:**
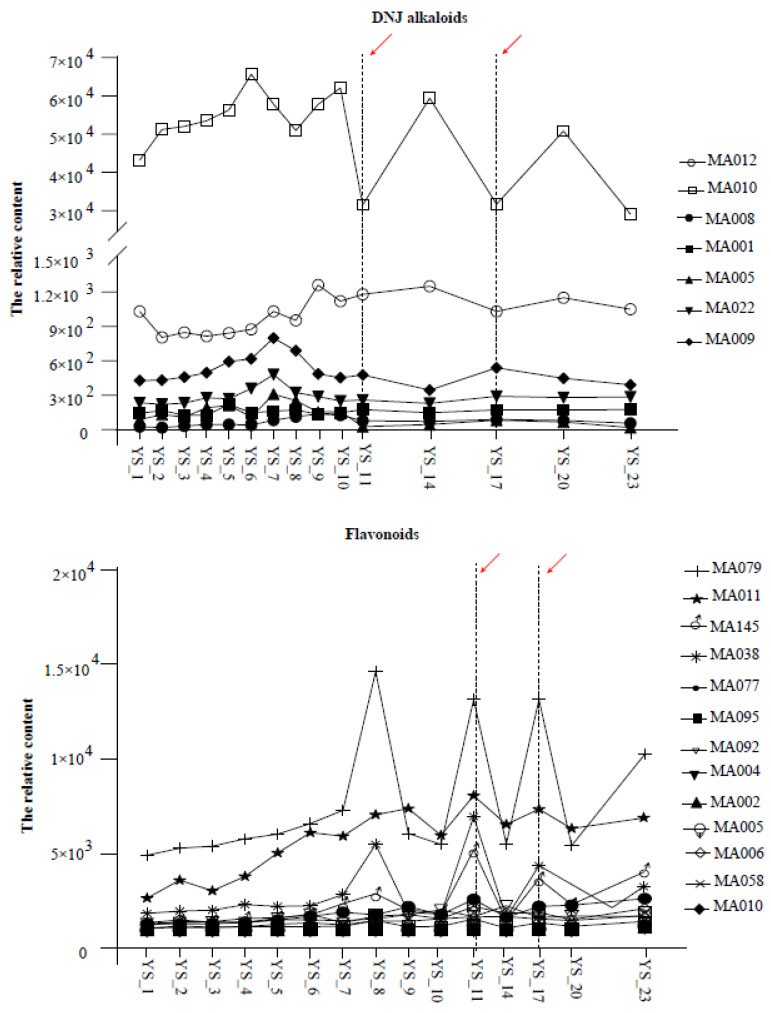
Accumulate patterns of DNJ alkaloid and flavonoids in *Morus nigra* after frost. The red arrows (dashed lines from left to right) from left to right represent the first and second descending frost, respectively.

To better understand the dynamic changes of key metabolites, we selected several metabolites with the most prominent fluctuations (CV > 100%) after frost for analysis. Results indicated that *Morus nigra* L. had a significantly higher content of DNJ alkaloids before frost compared to after. Furthermore, significant decreases were observed in some DNJ alkaloids (1,4-dideoxy-1,4-imino 4-imino-D-arabinitol and 4-Benzyl-1,4-dihydro-3-oxo-3*H*-pyrro [2,1-*c*] [1,4] oxazine-6-carboxaldehyde) on both the first and second days of frost (YS_11 and YS_17). Additionally, some flavonoids and their derivatives (kaempferol-3-*O*-rutinoside, Sanggenon T, 7,2′-Dihydroxy-4′-methoxy-8-prenylflavan, 2′, 4′, 7-Tri hydroxy-8-(2-hydroxyl) flavan 7-me) showed sharp increases on the same day of both frostings, with their contents being significantly higher after frosting than before (Figure 5). Consequently, we speculated on an inverse correlation between the dynamic changes of DNJ compounds and some flavonoid substances in *Morus nigra* L. Our correlation analysis further confirmed a significant negative correlation between DNJ alkaloids and certain flavonoids (Table 1).

**Figure 6:**
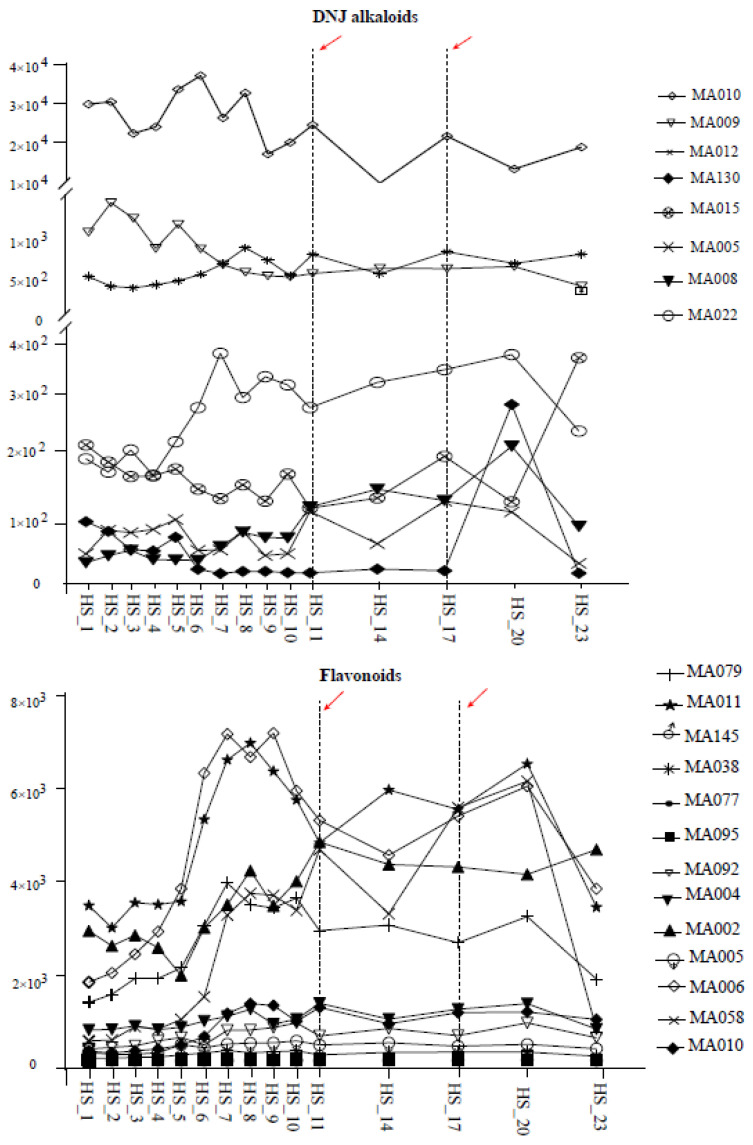
Accumulation patterns of DNJ alkaloids and flavonoids in *Morus nigra* L. after frost. The red arrows (dashed lines from left to right) indicate the first and second the frost from left to right respectively.

The dynamic changes of DNJ alkaloids in *Morus alba* L. exhibited a different pattern compared to *Morus nigra* L. Eight DNJ alkaloids showed significant increases after frost, with their levels continuously rising after the first frost, peaking after the second, and subsequently declining sharply. The dynamic change rule of flavonoids in *Morus alba* L. was also slightly different from that of *Morus nigra* L. Specifically, the content of flavonoids (13) increased sharply one week before the first frost, decreased significantly after the first frost, and then increased again until reaching its peak following the second frost (Figure 6).

Flavonoids are another essential bioactive compound in mulberry leaves and possess various health benefits, such as anti-bacterial, anti-inflammatory, lipid-lowering, glucose-lowering, and anti-oxidative abilities. Flavonoids have recently become a hot research topic in the investigation of the physiological activities of mulberry leaves as medicinal agents [17]. Yu’s study demonstrated that low temperatures following frost treatment could induce the expression of the *UFGT* gene in mulberry leaves, resulting in the accumulation of numerous flavonoid glycosides [12]. Similarly, Xu’s combined metabolomics and transcriptomics analyses indicated that several flavonoids were significantly enriched in the mulberry leaves of *Morus alba* L. under cold stress [13]. It has been found in some studies that seasonal changes can affect the levels of certain antioxidant metabolites in *Morus nigra* L. and *Morus alba* L. [14,17]. The findings of the present study are in agreement with the above-mentioned research, as the content of flavonoids in both mulberry leaves increased after passing through the first frost, with the highest level observed after the second frost.

DNJ has been recognized as a potent anti-hyperglycemic compound in mulberry leaves [18]. Research has indicated that the content of DNJ alkaloids exhibits a complex change trend during the growth and development of mulberry leaves [19]. Our study similarly found significant changes in DNJ content before and after frost. Interestingly, the changes in DNJ content in *Morus nigra* L. and *Morus alba* L. showed opposite trends before and after frost treatment. Specifically, the DNJ content in *Morus nigra* L. exhibited an overall decreasing trend after frost treatment. By contrast, DNJ content in *Morus alba* L. increased after the first frost, with a peak observed one day after the second frost. In a previous study, Kim et al. also reported significant differences in the genetic background among mulberry trees from various varieties, including differences in alkaloid content and change trends [20]. Therefore, we hypothesize that the content change trend of DNJ compounds after frost treatment may be influenced by not only the physiological state of mulberry leaf growth and development and environmental factors but also the differential enrichment responses of DNJ compounds in mulberry leaves of different varieties before and after frost treatment.

### 2.4. Metabolite Content in Mulberry Leaves Affected by the Day–Night Cycle

**Figure 7:**
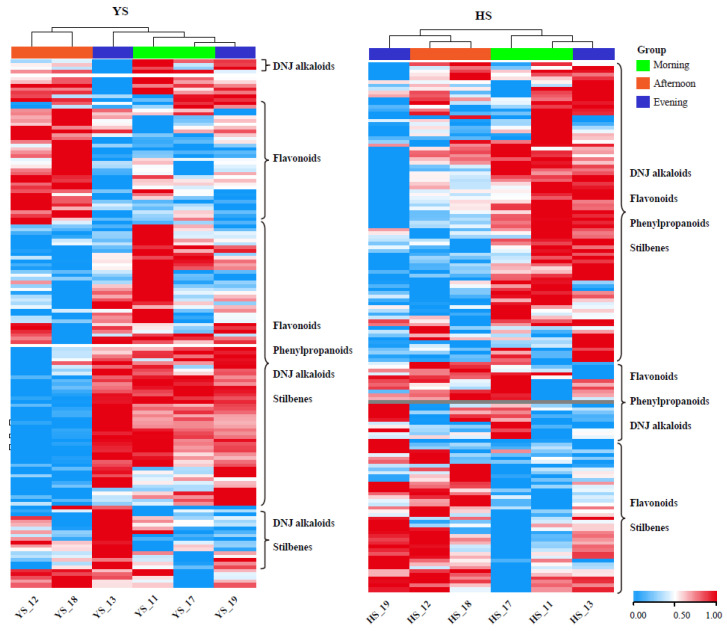
Effect of circadian rhythm on the accumulation patterns of *Morus nigra* L. and *Morus alba* L. metabolites.

To further investigate the influence of circadian rhythm on the metabolite composition of mulberry leaves, we collected leaf tissues from *Morus nigra* L. and *Morus alba* L. at three different time points: morning (9:00 a.m.), afternoon (4:00 p.m.), and evening (11:00 p.m.). The extracted metabolites were analyzed during the two frost treatments. As illustrated in Figure 7, the overall accumulation patterns for both *Morus nigra* L. and *Morus alba* L. were similar. Notably, significant differences in metabolite accumulation were observed between leaves collected in the afternoon versus those collected in the morning or at night. In *Morus nigra* L., with the exception of a small number of leaves collected at noon exhibiting high flavonoid content, most other substances, including DNJ alkaloids, flavonoids, phenylpropanoids, and stilbenes, were significantly lower than those in the morning and evening. Similar trends were found for *Morus alba* L. (Figure 7).

Marchetti et al. reported a positive correlation between the content of flavonols in mulberry leaves and the total hours of solar radiation on the picking day but a negative correlation with temperature [21]. Moreover, seasonal variations in DNJ content were found to be closely linked to temperature. Guo et al. reported that mulberry leaves after frost were significantly enriched in flavonoids. Furthermore, these leaves had the strongest ability to scavenge free radicals, which was significantly higher than those harvested in other seasons [22]. In our study, we also observed that the metabolite content in mulberry leaves was significantly influenced by the timing of leaf harvest on the picking date. Specifically, we analyzed the impact of circadian rhythms on metabolite accumulation in two varieties of mulberry leaves and found that harvesting in the morning yielded higher contents of DNJ alkaloids, flavonoids, and other substances. These results provide valuable information for optimizing the harvesting time of mulberry leaves to enhance their medicinal properties.

The studies mentioned above have highlighted the significant dynamic changes in metabolites in mulberry leaves after frost, which is of great importance for the rational use of their active compounds. Furthermore, these investigations have explored the rules governing the changes in metabolites in mulberry leaves before and after frost in depth. The findings not only enhance our understanding of the growth and developmental patterns of mulberry leaves but also provide essential theoretical support and guidance for their development and utilization. Further research should focus on elucidating the molecular mechanisms underlying the effects of environmental factors on mulberry plant metabolism and the biosynthesis of bioactive compounds, which could facilitate the development of novel strategies for improving the quality and quantity of mulberry-derived products.

## 3. Materials and Methods

### 3.1. Materials

Medicinal mulberry (*Morus nigra* L.) and black mulberry (*Morus alba* L.) were collected from Jiamu Fruit Science’s national long-term scientific research base in Xinjiang. Mulberry leaves were sampled every 7 days starting from 19 September 2021. When an early frost warning was issued, the sampling strategy was adjusted: samples were collected every three days for 15 days prior to and every day for 3 days leading up to the frost. Samples were taken once in the morning (9 a.m.), once in the afternoon (4 p.m.), and once in the evening (11 p.m.) on the day of the frost. After the frost, samples were collected daily for 3 consecutive days. Then, every 7 days, continuous sampling was carried out for 3 days before the second frost, consistent with the first frost sampling measure. All samples were collected by selecting three leaves (the 3rd-6th leaf from the top of the branch) from each tree, which were mixed and regarded as one biological repeat. The leaves were immediately frozen in liquid nitrogen and transferred to −80 °C after 1 h. Five trees were sampled, and the samples from each tree were stored separately. For metabolite detection, three biological repeats were selected. Unless otherwise specified, the sampling time was 9:00 a.m. (Table 2).

### 3.2. Experimental Methods

#### 3.2.1. Preparation and Extraction of Sample

Mulberry leaf samples were ground using a grinder (MM400, Retsch, Germany), after which 100 mg of powder was weighed. Next, 1 mL of a 70% methanol-0.5% formic acid water solution was added to the powder. The extraction was performed using an ultrasonic instrument (KQ-300DE, Kunshan, China) for 10 min. The extract was then placed in a low-temperature, high-speed centrifuge (MULTIFUGEXIR) and centrifuged at 10,000 r/min for 5 min. Then, 200 μL of supernatant was carefully collected into a 2 mL volumetric bottle. The chromatographic solution was filled to the scale with methanol and mixed using a 0.22 μm microporous filter membrane. Finally, the filtrate samples were stored in the sample bottle.

#### 3.2.2. Chromatographic and Mass Spectrometry Conditions

UPLC analysis was performed using an ACQUITY UPLC BEH C18 column (100 mm × 2.1 mm, 1.7 μm) on an I-Class system (Waters, USA). The column temperature was set at 45 °C, and the autosampler temperature was set at 10 °C. The mobile phase consisted of methanol (phase A) and a 0.1% formic acid aqueous solution (phase B). Gradient elution was carried out at a flow rate of 0.4 mL/min, with an injection volume of 2 μL. The gradient elution program was as follows: 0–3 min, 5–40% A; 3–10 min, 40–95% A; 10–12 min, 95% A; 12–13 min, 95–5% A; 13–15 min, 5% A.

For mass spectrometric analysis, a QTOF mass spectrometer (I-Class XEVO G2-XS, Waters, USA) equipped with an electrospray ion source (ESI) was used. The molecular weight scanning range was *m*/*z* 100–1200. The capillary voltage in positive/negative ion mode was set at 3.00 kV/2.50 kV, and the source and desolvation temperatures were 120 °C and 450 °C, respectively. The cone gas and desolvation gas flow rates were 50 L/h and 800 L/h, respectively, and the cone voltage was set to 40 V.

**Figure 8:**
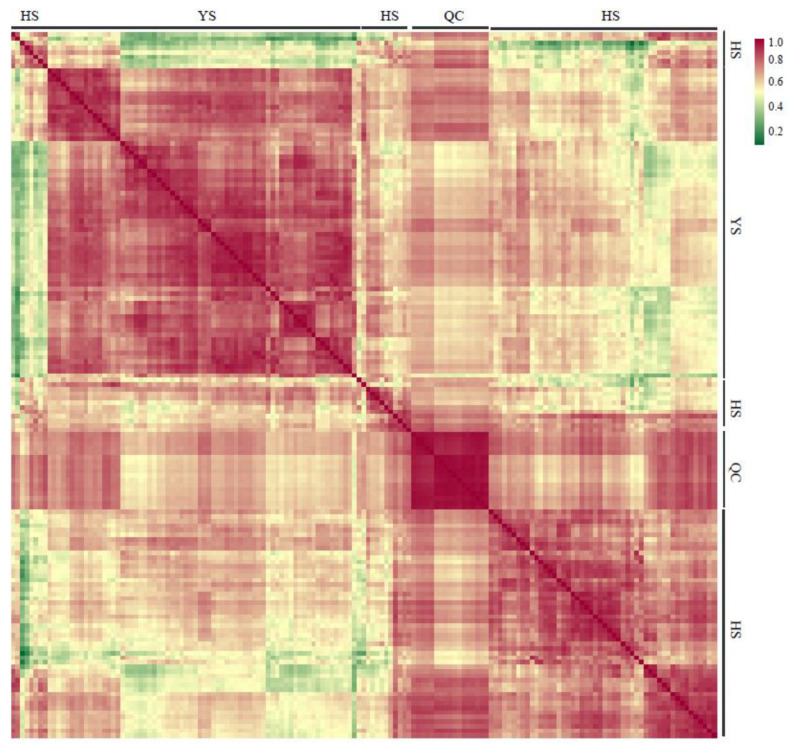
Repeated correlation quality of the control samples.

In MSE mode, the low-energy scan was not activated, and the energy for the high-energy scan was set to 15 eV–40 eV. To ensure quality, accuracy, and reproducibility, sodium formate was used as a calibration solution. Additionally, real-time mass number correction was performed using leucine enkephalin (LE) (*m*/*z* 556.2771 in positive ion mode and *m*/*z* 554.2615 in negative ion mode). A total of 17 quality control (QC) samples were prepared (Table S2). The QC samples were formulated by uniformly mixing the extractive solutions of all samples to assess whether the experimental conditions, such as instruments, were stable throughout the experiment. Correlations between the offline data of the metabolic group samples were analyzed to determine the biological repeat effect of the samples in the group. The higher the correlation coefficient, the stronger the biological repeat in the group and the more reliable the data. Pearson’s correlation coefficient (*r*) was used to evaluate the correlation index of biological repeat samples. Statistical results indicated that all 17 QC groups exhibited strong correlation with *r* values close to 1, confirming robust sample consistency within this experiment (Figure 8).

### 3.3. Metabolome Analysis

To screen differentially accumulated metabolites (DAMS), a threshold was applied, where DAMS were defined as metabolites exhibiting |log2 fold change (FC)|>1 and a variable importance in projection (VIP) value of at least 1. Subsequently, principal component analysis (PCA) was performed using SIMCA software version 14.1, with default settings.

## 4. Conclusions

The present study conducted a metabonomic analysis of different leaf tissues of *Morus nigra* L. and *Morus alba* L. before and after frost. Results showed significant differences in metabolite accumulation patterns between the two varieties before and after frost, indicating distinct types and contents of metabolites, which could lead to various applications. Additionally, the primary differential metabolites in the two varieties before and after frost were analyzed, and it was found that the DNJ content in *Morus nigra* L. showed an overall reducing trend after the frost, while flavonoids reached their peak during the second frost. For *Morus alba* L., DNJ increased after the first frost and peaked one day after the second frost. Flavonoids peaked mainly the week before the frost and again the day after the second frost, which suggests that frost had a more significant effect on *Morus nigra* L. To ensure the appropriate time for harvesting mulberry leaves with the target metabolites, it is recommended to consider the effects of frost and select the optimal harvest time accordingly. Finally, by analyzing the effect of circadian rhythm on metabolite accumulation in two varieties of mulberry leaves, it was concluded that harvesting mulberry leaves in the morning would result in higher contents of DNJ alkaloids, flavonoids, and other substances.

## Figures and Tables

**Table 1:** Correlations of DNJ alkaloids and flavonoid metabolites.

ID	Compounds	Class	DNJ					
			MA130	MA015	MA150	MA009	MA010	MA005
MA038	7,2′-Dihydroxy-4′-methoxy-8-prenylflavan	Flavonoids	−0.80	-	-	-	−0.53	−0.37
MA061	Alboctalol	Flavonoids	−0.36	−0.62	−0.33	-	−0.62	-
MA071	2′,4′,7-Trihydroxy-8-(2-hydroxyethyl)flavan 4-me-2-glc	Flavonoids	−0.84	-	-	-	-	−0.44
MA072	Kaempferol-3-*O*-rutinoside	Flavonoids	−0.44	−0.64	-	-	−0.62	-
MA080	Sanggenon T.	Flavonoids	−0.93	-	-	-	-	-
MA097	2′,4′,7-Trihydroxy-8-(2-hydroxyethyl)flavan 7-me	Flavonoids	−0.56	−0.55	−0.34	-	−0.62	-
MA099	cis-Mulberroside A.	Flavonoids	−0.65	−0.35	-	-	−0.59	−0.55
MA120	Isobavachin	Flavonoids	−0.37	−0.32	-	-	−0.73	-
MA123	Isobavachalcone	Flavonoids	−0.38	−0.39	-	-	−0.75	-
MA127	Sanggenofuran B	Flavonoids	−0.38	−0.59	−0.38	-	−0.65	-
MA133	Moracin I	Flavonoids	−0.52	-	-	-	−0.55	-
MA146	Apigenin-7-*O*-glucoside	Flavonoids	−0.51	−0.46	-	-	−0.62	−0.41
MA149	Sanggenofuran A.	Flavonoids	−0.61	-	-	-	-	-
MA126	Guangsangon I.	Flavonols	−0.34	−0.44	−0.41	-	−0.87	−0.52
MA135	Guangsangon B1	Flavonols	-	-	−0.39	-	−0.69	−0.50
MA024	Artoindonesianin O	Others	−0.46	−0.56	-	-	−0.61	-
MA103	Australone B	Others	−0.57	−0.41	−0.34	-	−0.76	−0.34
MA109	Wittifuran B	Others	-	−0.67	−0.36	-	−0.56	-
MA121	Yunanensin E	Others	−0.39	−0.61	−0.36	-	−0.79	−0.30
MA125	Yunanensin A	Others	−0.34	−0.43	−0.41	-	−0.79	−0.45
MA028	Ferulic acid	Phenylpropanoids	−0.52	-	-	-	−0.52	-
MA029	6,7-Dihydroxycoumarin	Phenylpropanoids	-	-	-	-	-	-
MA132	3′-*O*-[β-D-Glucopyranosyl-(1→6)-β-D-glucopyranoside], 4-*O*-β-D-glucopyranoside Mulberry	Polysaccharide	-	-	−0.44	−0.48	−0.54	−0.51
MA107	Mongolicin G.	Flavonoids	−0.51	-	-	−0.40	-	−0.38
MA119	Macrourin G	Flavonoids	-	−0.47	-	-	−0.72	-

**Table 2:** The sample collection timing of *Morus nigra* L. (YS) and *Morus alba* L. (HS) analyzed in the study.

Sampling Date	Time	HS_1	HS_2	HS_3	YS_1	YS_2	YS_2
9.19	9:00 a.m.	HS_1_1	HS_2_1	HS_3_1	YS_1_1	YS_2_1	YS_3_1
9.26	9:00 a.m.	HS_1_2	HS_2_2	HS_3_2	YS_1_2	YS_2_2	YS_3_2
9.29	9:00 a.m.	HS_1_3	HS_2_3	HS_3_3	YS_1_3	YS_2_3	YS_3_3
10.02	9:00 a.m.	HS_1_4	HS_2_4	HS_3_4	YS_1_4	YS_2_4	YS_3_4
10.05	9:00 a.m.	HS_1_5	HS_2_5	HS_3_5	YS_1_5	YS_2_5	YS_3_5
10.09	9:00 a.m.	HS_1_6	HS_2_6	HS_3_6	YS_1_6	YS_2_6	YS_3_6
10.13	9:00 a.m.	HS_1_7	HS_2_7	HS_3_7	YS_1_7	YS_2_7	YS_3_7
10.17	9:00 a.m.	HS_1_8	HS_2_8	HS_3_8	YS_1_8	YS_2_8	YS_3_8
10.21	9:00 a.m.	HS_1_9	HS_2_9	HS_3_9	YS_1_9	YS_2_9	YS_3_9
10.22	9:00 a.m.	HS_1_10	HS_2_10	HS_3_10	YS_1_10	YS_2_10	YS_3_10
10.23	9:00 a.m.	HS_1_11	HS_2_11	HS_3_11	YS_1_11	YS_2_11	YS_3_11
10.23	4:00 p.m.	HS_1_12	HS_2_12	HS_3_12	YS_1_12	YS_2_12	YS_3_12
10.23	4:00 p.m.	HS_1_13	HS_2_13	HS_3_13	YS_1_13	YS_2_13	YS_3_13
10.24	9:00 a.m.	HS_1_14	HS_2_14	HS_3_14	YS_1_14	YS_2_14	YS_3_14
10.24	4:00 p.m.	HS_1_15	HS_2_15	HS_3_15	YS_1_15	YS_2_15	YS_3_15
10.24	11:00 p.m.	HS_1_16	HS_2_16	HS_3_16	YS_1_16	YS_2_16	YS_3_16
10.25	9:00 a.m.	HS_1_17	HS_2_17	HS_3_17	YS_1_17	YS_2_17	YS_3_17
10.25	4:00 p.m.	HS_1_18	HS_2_18	HS_3_18	YS_1_18	YS_2_18	YS_3_18
10.25	11:00 p.m.	HS_1_19	HS_2_19	HS_3_19	YS_1_19	YS_2_19	YS_3_19
10.26	9:00 a.m.	HS_1_20	HS_2_20	HS_3_20	YS_1_20	YS_2_20	YS_3_20
10.26	4:00 p.m.	HS_1_21	HS_2_21	HS_3_21	YS_1_21	YS_2_21	YS_3_21
10.26	11:00 p.m.	HS_1_22	HS_2_22	HS_3_22	YS_1_22	YS_2_22	YS_3_22
10.30	9:00 a.m.	HS_1_23	HS_2_23	HS_3_23	YS_1_23	YS_2_23	YS_3_23

Note: The red color indicates frosty weather.

## Data Availability

The data that support the findings of this study are available from the corresponding author upon reasonable request.

## Supplementary Materials

The following supporting information can be downloaded at: https://www.mdpi.com/article/10.3390/molecules28124718/s1, Table S1: Metabolite Detection Information Table and CV Value Statistics of this Project; Table S2: Estimated LC-MS Peak Intensity of all Metabolites; Table S3: Differentially accumulation metabolites (DAMs) in HS before and after the First Frost; Table S4: Differentially accumulation metabolites (DAMs) in YS before and after the First Frost.
